# Clinical relevance of SARS-CoV-2 infection in late pregnancy

**DOI:** 10.1186/s12884-021-03985-1

**Published:** 2021-07-12

**Authors:** Marta Ruggiero, Edgardo Somigliana, Beatrice Tassis, Letizia Li Piani, Sara Uceda Renteria, Giussy Barbara, Giovanna Lunghi, Carlo Pietrasanta, Enrico Ferrazzi

**Affiliations:** 1 Department of Woman, New-Born and Child, Fondazione IRCCS Ca’ Granda Ospedale Maggiore Policlinico, Mangiagalli Centre, Via M. Fanti, 6, 20122 Milan, Italy; 2 Department of Clinical Sciences and Community Health, Università Degli Studi Di Milano, Milan, Italy; 3 Virology Unit, Fondazione IRCCS Ca’ Granda Ospedale Maggiore Policlinico, Mangiagalli Centre, Milan, Italy; 4 Neonatal Intensive Care Unit (NICU), Fondazione IRCCS Ca’ Granda Ospedale Maggiore Policlinico, Mangiagalli Centre, Milan, Italy

**Keywords:** Covid-19, Sars-Cov-2, Pregnancy, Antibodies

## Abstract

**Background:**

Evidence on the outcome of SARS-CoV-2 infection in pregnancy is generally reassuring but yet not definitive.

**Methods:**

To specifically assess the impact of SARS-CoV-2 infection in late pregnancy, we prospectively recruited 315 consecutive women delivering in a referral hospital located in Lombardy, Italy in the early phase of the epidemic. Restriction of the recruitment to this peculiar historical time period allowed to exclude infections occurring early in pregnancy and to limit the recall bias. All recruited subjects underwent a nasopharyngeal swab to assess the presence of Sars-Cov-2 using Real-time PCR. In addition, two different types of antibodies for the virus were evaluated in peripheral blood, those against the spike proteins S1 and S2 of the envelope and those against the nucleoprotein of the nucleocapsid. Women were considered to have had SARS-CoV-2 infection in pregnancy if at least one of the three assessments was positive.

**Results:**

Overall, 28 women had a diagnosis of SARS-CoV-2 infection in pregnancy (8.9%). Women diagnosed with the infection were more likely to report one or more episodes of symptoms suggestive for Covid-19 (*n* = 11, 39.3%) compared to unaffected women (*n* = 39, 13.6%). The corresponding OR was 4.11 (95%CI: 1.79–9.44). Symptoms significantly associated with Covid-19 in pregnancy included fever, cough, dyspnea and anosmia. Only one woman necessitated intensive care. Pregnancy outcome in women with and without SARS-CoV-2 infection did not also differ.

**Conclusions:**

SARS-CoV-2 infection is asymptomatic in three out of five women in late pregnancy and is rarely severe. In addition, pregnancy outcome may not be markedly affected.

## Background

The Severe Acute Respiratory Syndrome Coronavirus type-2 (SARS-Cov-2) was first identified in the area of Wuhan, China, at the end of 2019 but then rapidly spread globally in the beginning of 2020. In Lombardy, a region of Northern Italy that was particularly hit by the epidemic in the early phase, the first case was identified on February 20^th^, 2020 [1]. SARS-Cov-2 can potentially cause a fatal infection named Coronavirus disease 19 (Covid-19). Most common symptoms include fever, cough, sore throat, malaise, myalgia, gastrointestinal symptoms, anosmia and ageusia [2]. In more advanced conditions the subject can experience severe dyspnea and respiratory failure [3, 4].

Albeit still limited, there is evidence that SARS-Cov-2 can cause life-threatening situations also in pregnancy, even if fatal cases seem rare [5–13]. However, most evidence was obtained from case series or selected referral centers, a study design that could overestimate the detrimental effects of SARS-Cov-2. Indeed, asymptomatic or poorly symptomatic women are more likely to go undetected, thus boosting the epidemiological relevance of the infection. To assess the clinical relevance of SARS-Cov-2 infection in pregnancy one needs to know the real denominator, i.e. the total number of women being infected with the virus. Only population-based studies can protect the findings from this confounder [12, 13]. To note, even if very few, these type of studies tend to suggest that the rate of asymptomatic women could be higher than generally reported in clinical case series [12, 13].

The use of antibodies could provide more informative evidence on this issue because their presence could reveal a past infection also in asymptomatic cases. Two different types of antibodies can currently be tested, those against the glycoproteins of the virus envelope and those against the nucleoproteins. The former are generally considered neutralizing while the role of the latter in controlling virus replication is not yet established. Both were shown to be valuable markers of infection [14].

To shed more light on Covid-19 in pregnancy, we prospectively recruited consecutive women delivering in a large hospital located in Milan, Lombardy, in the early phase of the epidemic. All women underwent a nasopharyngeal swab to investigate the presence of the virus and provided a blood sample to assess the presence of antibodies against SARS-Cov-2. The primary aim was to determine the rate of pregnant women who were exposed to Covid-19 during pregnancy. Secondary aims included the clinical presentation of the infection and the possible impact on pregnancy outcome. To note, the recruitment period lasted from April 7^th^ to May 6^th^ and thus offered us the unique opportunity to study women who could be exposed to SARS-Cov-2 exclusively during the second part of pregnancy.

## Methods

The study took place at the Fondazione IRCCS Ca’ Granda Ospedale Maggiore Policlinico, a hospital located in Milan, in the Italian region of Lombardy. The hospital ensures about 5,000 deliveries per year and was committed by the local health and governmental officials to be a referral hospital also for Covid-19 in pregnancy. All women delivering between April 7^th^ to May 06^th^, 2020 were consecutively considered for study entry. Exclusion criteria were as follows: 1) pregnancy termination or miscarriage before 20 weeks’ gestation, 2) referral from other hospitals because of Covid-19 diagnosis. This criterion was introduced to prevent confounders due to referrals. Eligible women were invited to participate the day after delivery. Women agreeing to be recruited signed an informed consent. They were requested to fill a specific questionnaire during the time they spent in the hospital after delivery and provided a blood sample that was centrifuged at 3,500 rpm for 8 min at room temperature and stored at -20°Cuntil assayed for the presence of antibodies. For the nasopharyngeal swab for Sars-Cov-2, we relied on the ascertainment that was systematically performed at hospital admission. The study was approved by the local Institutional Review Board (Comitato Etico Milano Area 2, N. 356_2020) and we had the permission to collect the data from the hospital. All research was performed in accordance with the guidelines of the International Conference of Harmonization of Good Clinical Practice (ICH/GCP) and related regulations, including the Helsinki declaration of June 1964 and its adaptation done by the World Medical Association General Assembly in Seoul in 2008.

Collected information included general medical and obstetrics history as well as the pregnancy outcome of the index pregnancy. Patients’ charts were used to obtain this information. Obstetrics information were obtained as described elsewhere [15]. Small for gestational age (SGA) and large for gestational age (LGA) were defined as a newborn weight < 10 centile and > 90 centile, respectively. Centiles were determined using the local referral values [16]. The questionnaire that women had to fill evaluated the presence of symptoms suggestive for Covid-19 over the last three months. More than one episode of symptoms could be reported. The specific items included in the questionnaire are illustrated in details elsewhere [17]. Briefly, they include the presence of fever (≥ 37.5 °C), cough, sore throat, rhinitis, headache, diarrhea, vomit, dyspnea (including tachypnea), asthenia, myalgia, anosmia and ageusia. In addition, women were asked if they were exposed to higher risk of infection for job situations (health workers or laboratory technicians) or contacts with persons with a definitive diagnosis of Covid-19 or cohabitant with persons doing a job at risk or having symptoms suggestive for Covid-19.

Evaluation of Covid-19 in the cohort was based on three determinations: identification of the specific RNA on a nasopharyngeal swab and research of Immunoglobuline (Ig) using two different tests. Women were considered to have had Covid-19 in pregnancy if at least one of the assessments was positive The presence of the virus in the nasopharyngeal swab was investigated using the multiplex real-time (RT)-PCR for the qualitative detection of three target genes of SARS-CoV-2 (Allplex 2019-nCov Assay, Seegene, Seoul, Korea): SARS-CoV-2 nucleocapsid proteins and RNA polymerase gene fragments (both specific for Sars-Cov-2), and envelope gene fragment for detection of the sarbecovirus subgenus family that includes SARS-CoV-2. The cycle threshold (CT) used to define negativity was 40 cycles. The number of RT-PCR cycles needed to highlight positivity was calculated as the mean of those needed to indentify the three tested genes. The assay has a full-process negative control, positive control and internal control. The first serologic test was a chemiluminescence (CLIA) immunoassay (LIAISON®, Diasorin, Saluggia—Italy) used for the quantitative detection of Ig type G (IgG) antibodies against S1 and S2 antigens of SARS-CoV-2. The S1 and S2 proteins are derived from SARS-CoV-2 spike protein that is responsible for entry into the host cell. Antibodies against these two targets are neutralizing. The test has a sensitivity and specificity of 97.4% and 98.5%, respectively. The second test was an electrochemiluminescence (ECLIA) immunoassay (Elecsys®, Roche Diagnostics, Mannheim, Germany) that uses a recombinant nucleoprotein representing the nucleocapsid antigen for the qualitative determination of antibodies against SARS-CoV-2 (all antibodies, including IgG). This nucleoprotein antigen is internal to the envelope. The test has a specificity greater than 99.8% and a sensitivity of 100%.

Data analyses were performed using the Statistical Package for Social Sciences (SPSS 23.0, IL, USA). Women were considered to be affected if at least one of the three assessments resulted positive for Sars-Cov-2. The sample size (at least 250 subjects) was calculated expecting up to 20% of women being affected and aiming at a precision in the estimated prevalence of ± 5%. A binomial distribution model was used to determine the 95% Confidence Interval (CI) of the most relevant proportions. Data was compared using Student *t* Test, Fisher exact test or Chi Square test as appropriate. P values below 0.05 were considered statistically significant.

## Results

Three hundred eighty-five women delivered during the study period. Twelve were excluded because they were referred from other hospitals for Covid-19. Thirty-five were not recruited because of violation of the study protocol (the study was not proposed). Twenty-three women refused to participate. Three hundred fifteen women were ultimately enrolled. Overall, 28 women had a diagnosis of Sars-Cov-2 infection (8.9%, 95%CI: 6.2–12.5%), of whom 13 were identified with (RT)-PCR. The median (range) number of RT-PCR cycles needed to highlight positivity was 35.2 (13.5–39.7). Specific rates according to the test used are reported in Table 1. Details on the concordance among tests are illustrated in Table 2. To note, among the 24 women who were detected with antibodies against Sars-Cov-2, 17 (71%) were positive to both tests used, three (12%) were positive only for antibodies against the envelop and four (17%) only to those against the nucleoprotein. Baseline characteristics of women who did and did not have Sars-Cov-2 infection are shown in Table 3. The two groups did not significantly differ for any of these characteristics.

**Table 1:** Prevalence of women who had Covid-19 in pregnancy in the studied cohort (*n* = 315) according to type of testing

Diagnostic test	N	Rate	95%CI
Naso-pharyngeal swab RT-PCR	13	4.1%	2.4–6.9%
Antibodies againts CoV-2 envelope	20	6.4%	4.2–9.6%
Antibodies againts CoV-2 nucleoprotein	21	6.7%	4.4–10.0%
At least one	28	8.9%	6.2–12.5%

**Table 2:** Distribution of tests results among Covid-19 cases (*n* = 28)

Positive test			N (%)
RNA-PCR	Anti-envelope	Anti-nucleoprotein	
** + **	**-**	**-**	4 (14%)
**-**	** + **	**-**	3 (11%)
**-**	**-**	** + **	3 (11%)
** + **	** + **	**-**	0 (0%)
** + **	**-**	** + **	1 (4%)
**-**	** + **	** + **	9 (32%)
** + **	** + **	** + **	8 (28%)

**Table 3:** Baseline characteristics of women who did and did not test positive for Covid-19

Characteristics	Sars-Cov-2 infection	Controls	p
	*n* = 28	*n* = 287	
Age (years)	31.6 ± 7.0	34.2 ± 5.4	0.07
BMI (Kg/m^2^)	27.1 ± 4.5	26.4 ± 4.2	0.41
Gestational age (weeks)	38.8 ± 1.4	38.8 ± 2.0	0.79
Ethnicity			0.08
Caucasian	20 (71.4%)	230 (80.1%)	
Black / African	2 (7.2%)	26 (9.1%)	
Asian	0 (0.0%)	9 (3.1%)	
Hispanic	6 (21.4%)	22 (7.7%)	
Smoking in pregnancy	1 (3.6%)	12 (4.2%)	1.00
ART pregnancy	1 (3.6%)	22 (7.7%)	0.71
Previous vaginal deliveries	7 (25.0%)	89 (31.0%)	0.67
Previous cesarean sections	5 (17.9%)	45 (15.7%)	0.79
Previous myomectomy	0 (0.0%)	5 (1.7%)	1.00
Main medical complications			
Thyroid disorders	0 (0.0%)	22 (7.7%)	0.24
Autoimmune disorders	0 (0.0%)	9 (3.1%)	1.00
Cardiovascular problems	2 (7.2%)	6 (2.1%)	0.15
Thrombophylia	0 (0.0%)	3 (1.0%)	1.00
Respiratory disorders	0 (0.0%)	4 (1.4%)	1.00
Others	2 (7.2%)	7 (2.4%)	0.19
Multiple pregnancy	1 (3.6%)	10 (3.5%)	1.00
Flu vaccination in pregnancy	9 (32.1%)	96 (33.4%)	1.00

*ART* Assisted Reproductive Techniques

Eleven out of 28 women (39.3%) testing positive for Sars-Cov-2 reported one or more symptoms suggestive for Covid-19. Four had pneumonia, of whom three necessitated respiratory support. To note these four women were those becoming positive at RT-PCR with the lowest number of cycles (14.1, 13.5, 26.2 and 29.6).

Women diagnosed with the infection were more likely to report symptoms compared to unaffected women, the corresponding Odds Ratio (OR) being 4.11 (95%CI: 1.79–9.44) (Table 4). Symptoms significantly associated with Covid-19 included fever, cough, dyspnea and anosmia. A trend emerged also for ageusia (Table 4). In contrast, no difference emerged for conditions at higher risk of infection including type of job, reported direct contacts or cohabitation with persons at risk (Table 4).

**Table 4:** Symptoms and risk factors in women who did and did not test positive for Covid-19

Symptoms or risk conditions	Covid-19	Controls	p
	*n* = 28	*n* = 287	
Symptoms suggestive for Covid-19			
Fever	5 (17.9%)	13 (4.5%)	0.015
Cough	7 (25.0%)	19 (6.6%)	0.004
Sore throat	3 (10.7%)	17 (5.9%)	0.40
Rhinitis	4 (14.3%)	19 (6.6%)	0.14
Headache	0 (0.0%)	0 (0.0%)	n.a
Diarrhea	0 (0.0%)	2 (0.7%)	1.00
Vomit	1 (3.6%)	2 (0.7%)	0.24
Dyspnea	3 (10.7%)	0 (0.0%)	0.001
Asthenia	0 (0.0%)	2 (0.7%)	1.00
Myalgias	0 (0.0%)	2 (0.7%)	1.00
Anosmia	4 (14.3%)	1 (0.3%)	< 0.001
Ageusia	2 (7.1)	4 (1.4%)	0.09
Episodes of symptoms suggestive for Covid-19			0.001
None	17 (60.7%)	248 (86.5%)	
1	10 (35.7%)	38 (13.2%)	
2	1 (3.6%)	1 (0.3%)	
Risk conditions			
Job (health worker, lab technician)	0 (0.0%)	3 (1.0%)	1.00
Direct contact with Covid-19 affected persons	1 (3.6%)	3 (1.0%)	0.31
Cohabitant with persons at risk	3 (10.7%)	31 (10.8%)	1.00

Pregnancy outcome in women with and without Sars-Cov-2 infection is shown in Table 5. No statistically significant differences emerged. Only one woman with Sars-Cov-2 infection underwent cesarean delivery because of pneumonia and severe respiratory insufficiency. After delivery, she required admission in the intensive care unit. She has now recovered. No other women needed intensive care. No maternal deaths occurred. No SARS-Cov-2 neonatal infections at birth were observed.

**Table 5:** Pregnancy outcome in women who did and did not test positive for Covid-19

Characteristics	Sars-Cov-2 infection	Controls	p
	*n* = 28	*n* = 287	
Gestational Diabetes Mellitus	3 (10.7%)	22 (7.7%)	0.48
Hypertensive disorders	2 (7.1%)	6 (2.1%)	0.15
Intrahepatic cholestasis	1 (3.6%)	5 (1.7%)	0.43
Preterm delivery (< 37 weeks' gestation)	2 (7.1%)	22 (7.7%)	1.00
Cesarean section	10 (35.7%)	108 (37.6%)	1.00
SGA newborns ^a^	2 (7.4%)	30 (10.8%)	0.75
LGA newborns ^a^	1 (3.7%)	23 (8.3%)	0.71
Stilbirth	0 (0.0%)	0 (0.0%)	n.a
Early neonatal death	0 (0.0%)	0 (0.0%)	n.a
Maternal death	0 (0.0%)	0 (0.0%)	n.a
Apgar score at 1 min < 7	2 (7.1%)	8 (2.8%)	0.22
Neonatal pH < 7.10	2 (7.1%)	11 (3.8%)	0.32
NICU admission	5 (18%)	21 (7%)	0.07

For multiple pregnancies, data from the worse newborn was considered

*SGA* Small for gestational age, *LGA* Large for Gestational Age, *NICU* Neonatal Intensive Care Unit

^a^ Multiple pregnancies excluded

## Discussion

Sars-Cov-2 infection in pregnancy was not rare in our area during the first outbreak. One in 11 women (8.9%) actually entered in contact with the virus, less than estimated a priori. On the other hand, the clinical course of the disease appeared mostly unremarkable. Sixty-one percent did not report any symptom, preterm delivery because of Covid-19 maternal complications was necessary only in one case, and pregnancy outcome was not markedly influenced.

Interestingly, the rate of infected women observed in our study is very similar to the prevalence observed in a concomitant survey performed in our area and focusing on blood donors. Specifically, Valenti et al. evaluated the presence of antibodies against the nucleocapsid protein and reported for April 2020 a prevalence of 7.1% (95%CI: 4.4–10.8%), thus in line with our findings [18]. However, this prevalence is lower than hypothesized at the time of study design (when data from Valenti et al. was not yet available). This inconsistency is due to the use of mean regional data in the planning phase, while Sars-Cov-2 infection was distributed in patches. Milan downtown (where the study took place) was actually less touched in this first phase of the pandemic. This inaccuracy, however, did not affect the planned precision of the estimate (that was ± 5%). However, it limited the statistical power of the comparisons between women who did and did not have the infection.

In contrast, our reassuring clinical findings are somehow in disagreement with recent evidence from large case series of affected pregnant women. For instance, according to a recent systematic review of the literature, the rate of asymptomatic women was only 14.5%, 19% of affected women required delivery due to Covid-19 related reasons, 18.5% required oxygen support and preterm birth occurred in 21.5% of cases [7]. As already pointed out in the introduction, the most plausible explanation for the discrepancy with our findings is a selection bias. The denominator is radically different. By mainly focusing on the presence of antibodies and excluding referred cases, we were able to study an unselected population. In contrast, published case series reported on women who were mainly identified because of Covid-19 related symptoms. Our data could better reflect the real impact of Sars-Cov-2 infection in pregnancy. Of utmost relevance here is the very recent investigation from Crovetto et al. who also designed their study to protect their findings from selection biases [13]. The authors prospectively recruited women from three hospitals located in Spain during the same study period (March to May 2020) and excluded those from outside the catchment areas to avoid confounders. The rate of asymptomatic Sars-Cov-2 infections in pregnancy was 68.5%, thus in line with the 60.7% emerging from our analysis. This independent study strengthens the validity of our findings. Interestingly, a high rate of asymptomatic women was also suggested in the very initial study from Sutton et al. who performed the nasopharyngeal swab (but not antibodies) to 215 pregnant women at admission for delivery, and found that 87.9% of positive cases were asymptomatic [12]. Overall, one can reasonably infer that the infection does not cause symptoms in about two thirds of women, a much higher rate than what emerged from the meta-analysis (14.5%) [7]. Nonetheless, we cannot definitely rule out that differences in the characteristics of the population, local environmental conditions and genetic variants of the virus can impact on clinical relevance and could play a partial role in explaining these inconsistencies.

Some limitations of our study should be acknowledged. Firstly, the reliability of the diagnostic tests is still a source of debate. Even if Sars-Cov-2 infection was investigated using three different modalities, the accuracy of all the tests used is yet uncertain. The nasopharyngeal swab could detect only ongoing infections and the sensitivity in affected cases was reported to be only 63% [19]. Preliminary evidence is comforting for the other two tests employed to detect antibodies against Sars-Cov-2 but available studies for validation are not optimal. In particular, no attempts have yet been made to investigate the accuracy of these tests for asymptomatic or poorly symptomatic cases. Inferring results obtained in patients with moderate or severe forms of the disease to the whole population is arguable. More in general, the clinical and biological significance of the different type of antibodies remain to be ascertained [14, 20, 21]. Noteworthy, in our experience, agreement between the two tests was not excellent since only 17 women were found to be positive to both tests while 7 were positive only to one of the two. In addition, our test mainly evaluated IgG antibody (only one of the two kits included also other types of antibodies). Including precise evidence also for IgM and IgA could provide a more complete figure of the situation. On the other hand, misdiagnoses were unlikely considering that all women also performed the nasopharyngeal swab. Recent infections that could have not yet generated the immunological response were expected to be identified by the RT-PCR.

Secondly, even if women referred from other hospitals because of Covid-19 were excluded, we cannot rule out some other selection biases. On one hand, some healthy women with unremarkable history may have decided to deliver in other hospitals to avoid an Institution with Covid-19 affected cases while, on the other hand, some women with mild symptoms suggestive for the infection could have been more likely to refer to our hospital. Both biases could lead to overestimate the detected frequency of Covid-19. However, the high proportion of asymptomatic cases tends to rule out a major role of these confounders.

Finally, since Covid-19 related symptoms were retrospectively collected after delivery, one cannot exclude a recall bias. In this regard, it has however to be underlined that women were blinded to the results of the antibodies tests when interviewed and that the investigated period of time was limited to only three months. Even if episodes of mild symptoms could be overlooked, it is unlikely that more significant health troubles could be omitted.

## Conclusions

Women in late pregnancy do not appear to be more susceptible to Covid-19; the observed prevalence overlaps with the non-pregnant population of the same area. In addition, the study suggests that the course of the disease in late pregnancy is unremarkable in the majority of cases.

## Data Availability

The database can be provided on request.

## Abbreviations

ART: Assisted Reproductive Technique
CI: Confidence Interval
CT: Cycle Threshold
CLIA: Chemiluminescence
Covid-19: Coronavirus Infection Disease – 19
ECLIA: Electrochemiluminescence
ICH/GCP: International Conference of Harmonization of Good Clinical Practice
IgG: Immunoglobuline-type G
IRCCS: Istituto di Ricerca e Cura a Carattere Scientifico
OR: Odds Ratio
RT-PCR: Multiplex Real-Time in Polymerase Chain Reaction
SARS-Cov-2: Severe Acute Respiratory Syndrome Coronavirus type-2
